# Novel nonsense variant of *KIF11* in a patient with MCLMR

**DOI:** 10.1038/s41439-026-00341-7

**Published:** 2026-03-02

**Authors:** Yuko Ozaki, Kyoko Yokoi, Yasuhisa Nakamura, Masanori Fujimoto, Risako Ishioka, Kozue Kasukabe, Takenori Kato, Shinji Saitoh

**Affiliations:** 1 Department of Pediatrics, Nagoya City University West Medical Center, Nagoya, Japan; 2 Department of Pediatrics and Neonatology, Nagoya City University Graduate School of Medical Sciences, Nagoya, Japan

**Keywords:** Neurological disorders, Disease genetics

## Abstract

Microcephaly with or without chorioretinopathy, lymphedema or mental retardation is a rare KIF11-related disorder. Here we report the case of a patient with microcephaly, lymphedema, nystagmus and familial exudative vitreoretinopathy carrying a novel de novo *KIF11* nonsense variant (NM_004523.4:p.Glu123Ter), which is considered pathogenic. This case expands the phenotypic range of *KIF11* pathogenic variants and highlights the importance of early ophthalmological evaluation, genetic counseling and family assessment.

Microcephaly with or without chorioretinopathy, lymphedema or mental retardation (MCLMR) is a rare autosomal dominant disorder characterized by congenital microcephaly, lymphedema and chorioretinal dysplasia, and is associated with pathogenic variants of the kinesin family member 11 (*KIF11*) gene^1,2^. The prevalence of MCLMR is estimated to be less than 1 in 1,000,000 people worldwide (https://www.orpha.net, accessed 12 December 2025). *KIF11* is a mitotic motor protein and a member of the kinesin superfamily. *KIF11* slides along microtubules during cell division and contributes to the maintenance of spindle architecture. Loss of *KIF11* function has been reported to cause mitotic abnormalities and apoptotic cell death, which ultimately contribute to the loss of neural progenitor cells and small brain size^3^. *KIF11* has been established as one of the causative genes for familial exudative vitreoretinopathy^4,5^.

The patient was the first male child of unrelated parents, born at 39 weeks’ gestation via normal delivery. The pregnancy resulted from in vitro fertilization, and the patient’s family history was unremarkable. His height was 49.0 cm (−0.14 standard deviation (s.d.)), weight was 2,876 g (−0.54 s.d.) and head circumference was 31.0 cm (−1.75 s.d.). Edema was observed on the dorsum of the feet from birth and did not improve (Fig. 1A). At 3 months of age, his height was 61.4 cm (0 s.d.), weight was 5,090 g (−1.63 s.d.) and head circumference was 35.0 cm (−3.66 s.d.). At this age, horizontal nystagmus was observed, and microcephaly progressed (Fig. 1B). Routine laboratory tests and head magnetic resonance imaging (MRI) revealed no abnormalities except for microcephaly (Fig. 1C). At 5 months of age, bilateral retinal folds were observed, leading to a familial exudative vitreoretinopathy diagnosis (Fig. 1D). He could hold his head up at 4 months of age, roll over at 6 months, sit by himself at 8 months, stand at 1 year 2 months and walk at 1 year 4 months. He could also speak more than five words at 1 year 7 months of age. So far, no developmental delays have been reported. Due to his young age, a formal assessment of vision could not be performed. He appeared to see large distant objects but had difficulty seeing nearby objects.

**Fig. 1:**
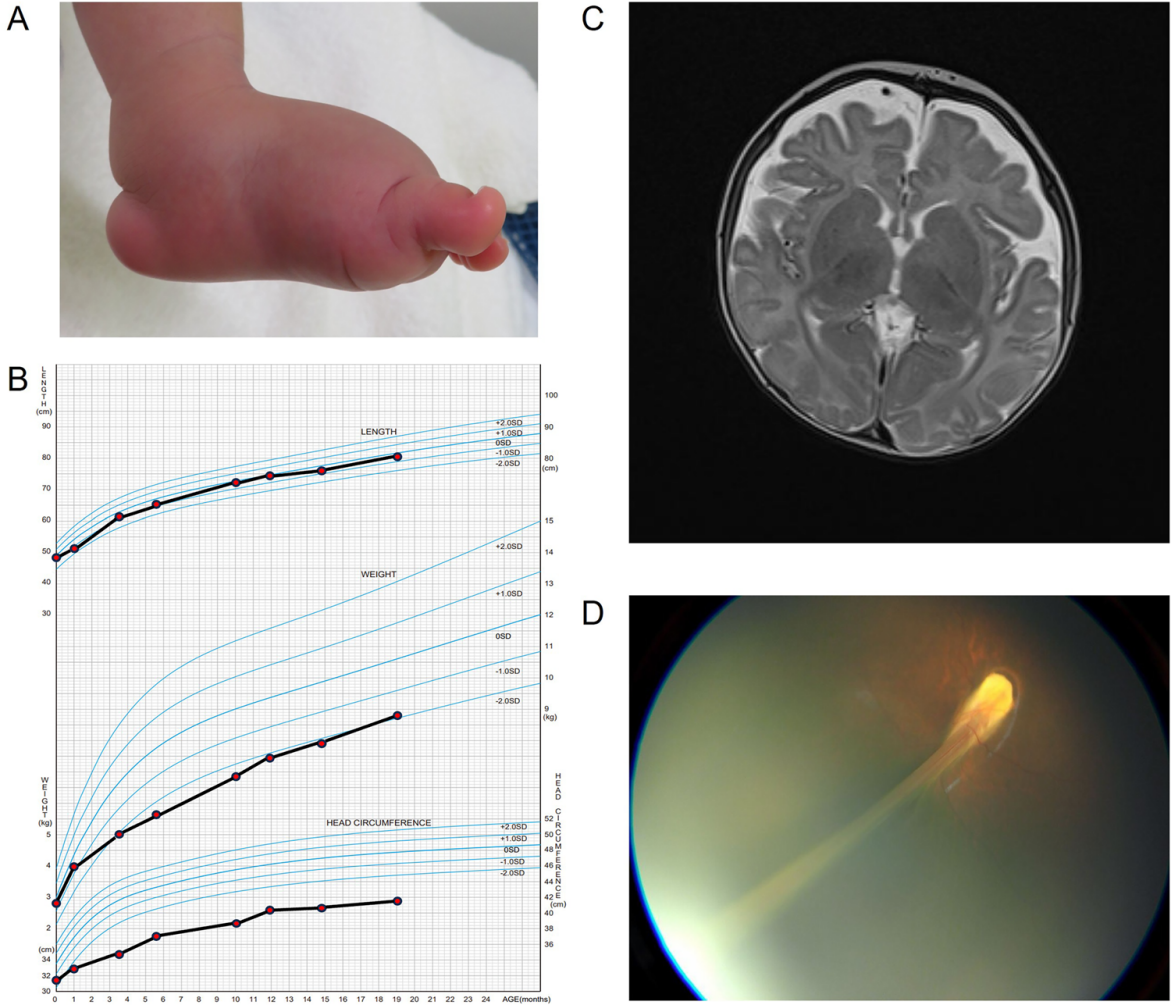
Clinical features of the patient. **A** Lymphedema on the feet at 1 month of age. **B** Cross-sectional growth chart for age^9,10^. **C** Head MRI findings at 3 months of age. **D** Retinal fold shown by fundus photograph at 5 months of age.

Genomic DNA was extracted from the peripheral blood using standard procedures, and informed consent was obtained from the patient’s parents. Exome sequencing was performed using the NovaSeq 6000 Sequencing System (Illumina). This analysis identified a heterozygous nonsense variant in *KIF11*, NM_004523.4:c.367G>T p.(Glu123Ter), which has not been reported in the Genome Aggregation Database (gnomAD; https://gnomad.broadinstitute.org/). Subsequent Sanger sequencing of the patient and his parents confirmed that the variant arose de novo (Fig. 2). The patient’s clinical features were consistent with the known phenotype associated with *KIF11* variants. According to the American College of Medical Genetics and Genomics guidelines, this nonsense variant is classified as a pathogenic variant (PVS1, null variant; PM2, absent from population databases; PP4, phenotype specificity)^6^.

**Fig. 2:**
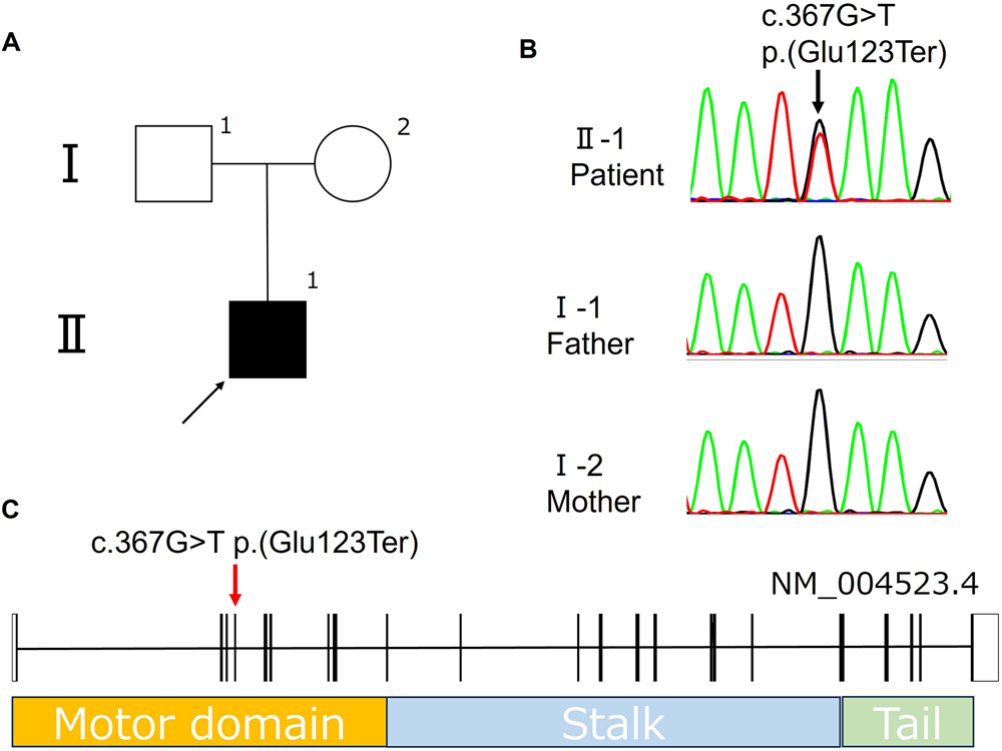
Pedigree and genetic features of the studied family. **A** Pedigree of the studied family. Solid symbols: affected individuals; open symbols: unaffected individuals. The proband is indicated with an arrow. **B** Sanger sequencing confirmed a missense variant (c.367G>T p.Glu123Ter) in *KIF11*; it was present in the proband but not detected in either parent. **C** The genomic structure of *KIF11*. The nonsense variant NM_004523.4:c.367G>T (p.Glu123Ter) is indicated by a red arrow.

The clinical presentation of our patient included chorioretinopathy, congenital microcephaly and dorsal pedal lymphedema and is consistent with the well-described phenotype of MCLMR^1,2^. In clinical practice, chorioretinopathy in older children is often detected only after the onset of strabismus or amblyopia. Conversely, infants may initially present with nystagmus, which can serve as an important early indicator. Given that pediatricians may not routinely conduct detailed eye examinations, nystagmus may be an important sign that warrants prompt ophthalmologic consultation, thereby facilitating the diagnosis of chorioretinopathy.

MCLMR is known to exhibit very little genotype–phenotype correlation^1^. Variants in *KIF11* have been associated with a wide clinical spectrum, ranging from severe retinal and neurodevelopmental abnormalities to markedly attenuated or even subclinical presentations. Our patient showed a typical clinical presentation of MCLMR. The *KIF11* variant is expected to have induced protein truncation in the motor domain located close to the N terminal, thus indicating null function from the variant allele^7^. Notably, pathogenic variants, including truncating variants, have been identified in asymptomatic parents^1^, indicating incomplete penetrance and underscoring the variability of clinical expression. In addition, parental mosaicism has been reported in rare cases^8^, further contributing to the heterogeneity of inheritance patterns. Although the disorder follows an autosomal dominant inheritance pattern, approximately 40% of cases are caused by de novo variants^1^, reflecting the diverse mechanisms underlying this disorder’s presentation.

In our patient, the variant was confirmed to be de novo, a valuable finding for genetic counseling because it indicates a low risk of recurrence in future pregnancies. Determining the origin of the variant also clarified the inheritance pattern in this family, which is an important consideration in KIF11-related disorders, due to their marked phenotypic variability. Given this variability and the documented occurrence of asymptomatic or minimally affected carriers, parental evaluation is essential. This evaluation is critical not only for accurate diagnosis, but also for providing precise genetic counseling and for further delineating the full clinical spectrum associated with *KIF11* variants.

This case may ultimately aid in refining genotype–phenotype correlations in KIF11-related disorders and offer insights into the biological mechanisms involved.

## HGV database

The relevant data from this Data Report are hosted at the Human Genome Variation Database at 10.6084/m9.figshare.hgv.3607.
